# 3,3′-(*p*-Phenyl­ene)bis­(3,4-dihydro-2*H*-1,3-benzoxazine)

**DOI:** 10.1107/S1600536809005790

**Published:** 2009-02-25

**Authors:** Sekaran Ranjith, Sundar Thenmozhi, Ramaiyan Manikannan, Shanmugam Muthusubramanian, Arunachalathevar Subbiahpandi

**Affiliations:** a Department of Physics, Presidency College (Autonomous), Chennai 600 005, India; b Department of Organic Chemistry, School of Chemistry, Madurai Kamaraj University, Madurai 625 021, India

## Abstract

Mol­ecules of the title compound, C_22_H_20_N_2_O_2_, are situated on crystallographic centres of symmetry. The oxazinane ring adopts a sofa conformation. Mol­ecules are linked into cyclic centrosymmetric dimers *via* C—H⋯O hydrogen bonds with the motif *R*_2_^2^(6). In addition to the C—H⋯O inter­actions, the crystal structure is also stabilized by C—H⋯π inter­actions.

## Related literature

For related structures, see: Huerta *et al.* (2006 ▶). For the biological activity of bis-benzoxazine compounds, see: Billmann & Dorman (1963 ▶); Heinisch *et al.* (2002 ▶). For puckering and asymmetry parameters, see: Cremer & Pople (1975 ▶); Nardelli (1983 ▶).
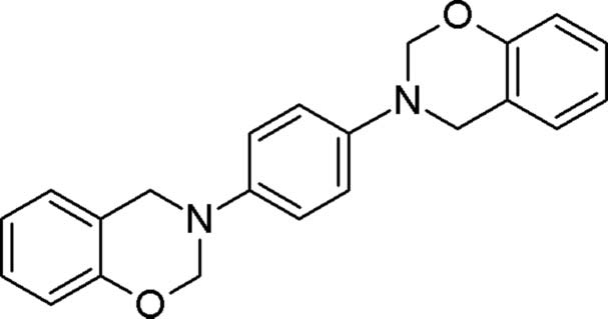


## Experimental

### 

#### Crystal data

C_22_H_20_N_2_O_2_
*M* *_r_* = 344.40
Monoclinic, 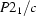

*a* = 9.191 (5) Å
*b* = 8.794 (4) Å
*c* = 11.317 (5) Å
β = 113.90 (3)°
*V* = 836.3 (7) Å^3^
*Z* = 2
Mo *K*α radiation
μ = 0.09 mm^−1^
*T* = 293 K
0.21 × 0.19 × 0.16 mm

#### Data collection

Bruker APEXII CCD area-detector diffractometer
Absorption correction: multi-scan (*SADABS*; Sheldrick, 1996 ▶) *T*_min_ = 0.982, *T*_max_ = 0.986
13095 measured reflections
3707 independent reflections
2762 reflections with *I* > 2σ(*I*)
*R*_int_ = 0.021

#### Refinement

*R*[*F* ^2^ > 2σ(*F* ^2^)] = 0.050
*wR*(*F* ^2^) = 0.164
*S* = 1.02
3707 reflections
118 parameters
H-atom parameters constrained
Δρ_max_ = 0.42 e Å^−3^
Δρ_min_ = −0.21 e Å^−3^

### 

Data collection: *APEX2* (Bruker, 2004 ▶); cell refinement: *APEX2*; data reduction: *SAINT* (Bruker, 2004 ▶); program(s) used to solve structure: *SHELXS97* (Sheldrick, 2008 ▶); program(s) used to refine structure: *SHELXL97* (Sheldrick, 2008 ▶); molecular graphics: *ORTEP-3* (Farrugia, 1997 ▶); software used to prepare material for publication: *SHELXL97* and *PLATON* (Spek, 2009 ▶).

## Supplementary Material

Crystal structure: contains datablocks global, I. DOI: 10.1107/S1600536809005790/fb2131sup1.cif

Structure factors: contains datablocks I. DOI: 10.1107/S1600536809005790/fb2131Isup2.hkl

Additional supplementary materials:  crystallographic information; 3D view; checkCIF report

## Figures and Tables

**Table 1:** Hydrogen-bond geometry (Å, °)

*D*—H⋯*A*	*D*—H	H⋯*A*	*D*⋯*A*	*D*—H⋯*A*
C8—H8*A*⋯O1^i^	0.97	2.49	3.3469 (15)	147
C3—H3⋯*Cg*1^ii^	0.93	2.72	3.582 (13)	155
C3—H3⋯*Cg*1^iii^	0.93	2.72	3.582 (13)	155

Symmetry codes: (i) 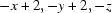; (ii) 
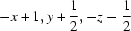
; (iii) 
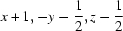
. *Cg*1 is the centroid of the C9–C11/C9a–C11a ring.

**Fig. 1.**
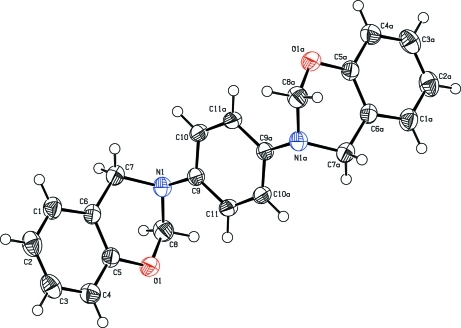
View of the title molecule with the atom labelling scheme. The displacement ellipsoids are drawn at the 30% probability level while the H atoms are shown as small spheres of arbitrary radii.

**Fig. 2.**
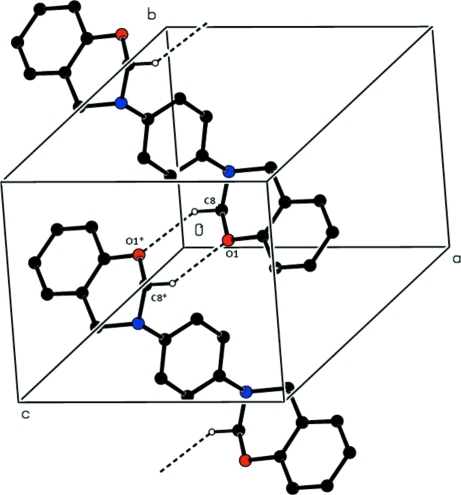
The crystal structure showing the centrosymmetric hydrogen bond motif R22(6). For the sake of clarity, the H atoms not involved in the motif have been omitted. The atoms marked with an asterisk (*) are situated in the position (2-x, 2-y, -z). The dashed lines indicate the hydrogen bonds.

## supplementary crystallographic information

### Comment

Bis-benzoxazine compounds exhibit various biological activities including antibacterial (* i. a. * tuberculostatic), antitumor, fungicidal and plant-growth regulative properties (Billmann & Dorman, 1963; Heinisch *et al.*, 2002). Polyoxymethylene (paraformaldehyde) undegoes a bimolecular condensation with *N*,*N*-bis-(*o*-hydroxybenzyl)-ethylenediamine in benzene to form 1,2-bis-[3-(3,4-dihydro-1,3–2*H*-benzoxazino]-ethane, which shows bacteriostatic and fungistatic activities (Billmann & Dorman, 1963). Taking into consideration these aspects, and in order to obtain a detailed information on the molecular structure in the solid state, the X-ray structure determination of the title compound has been carried out.

The molecules are situated on the crystallographic centres of symmetry and therefore have symmetry 1 (Fig. 1). The bond lengths N1—C7, O1—C5 are normal and comparable to the corresponding values observed in the related structure of 1,4-bis(8-tert-butyl-6-methyl-4*H*-1,3-benzoxazin-3-yl)benzene (Huerta *et al.*, 2006).

The oxazinane ring of the benzoxazine moiety adopts the sofa conformation, with the puckering parameters q_2_ and φ (Cremer & Pople, 1975) and the smallest displacement asymmetric parameters, Δ_s_ (Nardelli *et al.*, 1983) as follows: q_2_=0.3657 (11) Å, φ=264.71 (17)°, Δ_s_(N1)=21.40 (11).

In addition to the van der Waals interactions, the crystal packing is stabilized by C–H···O and C–H···π hydrogen bonds (Tab. 1) as well as by π–π-electron interactions. The atom C8 acts as a donor to the atom O1 of the neighbour molecule. This hydrogen bond is involved in a motif *C*_1_^1^(10) forming an infinite chain along *b* axis. *C*_1_^1^(10) projected on the axis *b* corresponds to the translational period along this axis. Simultaneously a pair of C8–H8A···O1 hydrogen bonds form a cyclic centrosymmetric dimer [*R*_2_^2^(6)]. The π–π-electron interactions between the rings (C1\C2···C6) at *x*, *y*, *z* and 1-*x*, -*y*, -*z* with the centroid-centroid distances equal to 3.780 (2) Å are observed in the crystal structure.

### Experimental

A mixture of 2-(4-[(2-hydroxybenzyl)amino]anilinomethyl)benzenol (0.005 mole) and methanal (0.010 mole) was irradiated under microwaves generated by IFB Microwave Oven (Frequency = 2450 MHz ~λ=122 mm; 480 W) for 4 to 6 minutes; the distance from the source to the sample was 15 cm. The progress of the reaction was monitored by a thin layer chromatography. After completion of reaction, ice-cold water (50 ml) was added to the reaction mixture and stirred. The title compound was extracted with chloroform, the combined organic layers were dried over anhydrous sodium sulfate and then the solvent was evaporated. The crude product was recrystallized in ethylacetate at room temperature. The elongated single cystals of the title compound of average length of 2 mm were grown.

### Refinement

All the H atoms could be clearly discerned in the difference electron density map. Nevertheless the atoms were situated into the idealized positions and refined in the riding model approximation. The constraints: C_aryl_-H = 0.93 and C_methylene_-H = 0.97 Å. U_iso_H=1.2U_eq_(C_aryl_/_methylene_).

### Crystal data

**Table:**

C_22_H_20_N_2_O_2_	*F*(000) = 364
*M**_r_* = 344.40	*D*_x_ = 1.368 Mg m^−^^3^
Monoclinic, *P*2_1_/*c*	Mo *K*α radiation, λ = 0.71073 Å
Hall symbol: -P 2ybc	Cell parameters from 3779 reflections
*a* = 9.191 (5) Å	θ = 2.4–35.3°
*b* = 8.794 (4) Å	µ = 0.09 mm^−^^1^
*c* = 11.317 (5) Å	*T* = 293 K
β = 113.90 (3)°	Block, colourless
*V* = 836.3 (7) Å^3^	0.21 × 0.19 × 0.16 mm
*Z* = 2	

### Data collection

**Table:**

Bruker APEXII CCD area-detector diffractometer	3707 independent reflections
Radiation source: fine-focus sealed tube	2762 reflections with *I* > 2σ(*I*)
graphite	*R*_int_ = 0.021
ω scans	θ_max_ = 35.3°, θ_min_ = 2.4°
Absorption correction: multi-scan (*SADABS*; Sheldrick, 1996)	*h* = −14→13
*T*_min_ = 0.982, *T*_max_ = 0.986	*k* = −14→14
13095 measured reflections	*l* = −18→16

### Refinement

**Table:**

Refinement on *F*^2^	Primary atom site location: structure-invariant direct methods
Least-squares matrix: full	Secondary atom site location: difference Fourier map
*R*[*F*^2^ > 2σ(*F*^2^)] = 0.050	Hydrogen site location: difference Fourier map
*wR*(*F*^2^) = 0.164	H-atom parameters constrained
*S* = 1.02	*w* = 1/[σ^2^(*F*_o_^2^) + (0.092*P*)^2^ + 0.1065*P*] where *P* = (*F*_o_^2^ + 2*F*_c_^2^)/3
3707 reflections	(Δ/σ)_max_ < 0.001
118 parameters	Δρ_max_ = 0.42 e Å^−^^3^
0 restraints	Δρ_min_ = −0.21 e Å^−^^3^
40 constraints	

### Special details

**Table:**

Geometry. All e.s.d.'s (except the e.s.d. in the dihedral angle between two l.s. planes) are estimated using the full covariance matrix. The cell e.s.d.'s are taken into account individually in the estimation of e.s.d.'s in distances, angles and torsion angles; correlations between e.s.d.'s in cell parameters are only used when they are defined by crystal symmetry. An approximate (isotropic) treatment of cell e.s.d.'s is used for estimating e.s.d.'s involving l.s. planes.
Refinement. Refinement of *F*^2^ against ALL reflections. The weighted *R*-factor *wR* and goodness of fit *S* are based on *F*^2^, conventional *R*-factors *R* are based on *F*, with *F* set to zero for negative *F*^2^. The threshold expression of *F*^2^ > σ(*F*^2^) is used only for calculating *R*-factors(gt) *etc*. and is not relevant to the choice of reflections for refinement. *R*-factors based on *F*^2^ are statistically about twice as large as those based on *F*, and *R*- factors based on ALL data will be even larger.

### Fractional atomic coordinates and isotropic or equivalent isotropic displacement parameters (Å^2^)

**Table:**

	*x*	*y*	*z*	*U*_iso_*/*U*_eq_	
C1	0.43532 (12)	0.74937 (13)	−0.05691 (12)	0.0396 (2)	
H1	0.4025	0.6810	−0.0100	0.048*	
C2	0.32355 (12)	0.81402 (14)	−0.16802 (13)	0.0455 (3)	
H2	0.2166	0.7884	−0.1960	0.055*	
C3	0.37168 (13)	0.91652 (15)	−0.23690 (12)	0.0450 (3)	
H3	0.2968	0.9594	−0.3121	0.054*	
C4	0.52981 (13)	0.95635 (13)	−0.19555 (11)	0.0407 (2)	
H4	0.5613	1.0267	−0.2418	0.049*	
C5	0.64179 (11)	0.89038 (11)	−0.08403 (10)	0.03299 (19)	
C6	0.59569 (11)	0.78485 (11)	−0.01425 (10)	0.03196 (19)	
C7	0.71934 (12)	0.71327 (13)	0.10586 (11)	0.0380 (2)	
H7A	0.7147	0.7601	0.1819	0.046*	
H7B	0.6966	0.6058	0.1075	0.046*	
C8	0.89981 (12)	0.88699 (12)	0.08050 (12)	0.0395 (2)	
H8A	1.0095	0.9023	0.0928	0.047*	
H8B	0.8796	0.9520	0.1415	0.047*	
C9	0.93646 (9)	0.61784 (10)	0.05017 (9)	0.02906 (18)	
C10	0.93371 (10)	0.46677 (11)	0.08570 (9)	0.03247 (19)	
H10	0.8888	0.4429	0.1437	0.039*	
C11	1.00414 (11)	0.64904 (11)	−0.03736 (10)	0.03289 (19)	
H11	1.0076	0.7487	−0.0636	0.039*	
N1	0.87985 (9)	0.73251 (10)	0.10900 (8)	0.03406 (18)	
O1	0.79736 (9)	0.93289 (9)	−0.04855 (8)	0.0422 (2)	

### Atomic displacement parameters (Å^2^)

**Table:**

	*U*^11^	*U*^22^	*U*^33^	*U*^12^	*U*^13^	*U*^23^
C1	0.0309 (4)	0.0399 (5)	0.0495 (6)	−0.0034 (4)	0.0178 (4)	−0.0027 (4)
C2	0.0291 (4)	0.0477 (6)	0.0531 (6)	−0.0014 (4)	0.0098 (4)	−0.0076 (5)
C3	0.0367 (5)	0.0511 (6)	0.0380 (5)	0.0088 (4)	0.0058 (4)	−0.0019 (4)
C4	0.0410 (5)	0.0437 (5)	0.0385 (5)	0.0057 (4)	0.0172 (4)	0.0055 (4)
C5	0.0296 (4)	0.0340 (4)	0.0369 (5)	0.0001 (3)	0.0151 (3)	−0.0001 (3)
C6	0.0282 (4)	0.0338 (4)	0.0357 (4)	0.0001 (3)	0.0149 (3)	−0.0011 (3)
C7	0.0336 (4)	0.0450 (5)	0.0392 (5)	0.0028 (4)	0.0188 (4)	0.0064 (4)
C8	0.0331 (4)	0.0361 (5)	0.0458 (6)	−0.0037 (3)	0.0121 (4)	−0.0023 (4)
C9	0.0211 (3)	0.0341 (4)	0.0281 (4)	−0.0013 (3)	0.0059 (3)	0.0035 (3)
C10	0.0287 (4)	0.0372 (4)	0.0319 (4)	−0.0008 (3)	0.0126 (3)	0.0071 (3)
C11	0.0294 (4)	0.0329 (4)	0.0359 (4)	−0.0010 (3)	0.0128 (3)	0.0076 (3)
N1	0.0281 (3)	0.0366 (4)	0.0361 (4)	−0.0003 (3)	0.0115 (3)	0.0012 (3)
O1	0.0308 (3)	0.0426 (4)	0.0526 (5)	−0.0023 (3)	0.0163 (3)	0.0117 (3)

### Geometric parameters (Å, °)

**Table:**

C1—C2	1.3832 (16)	C7—H7A	0.9700
C1—C6	1.3879 (13)	C7—H7B	0.9700
C1—H1	0.9300	C8—N1	1.4252 (13)
C2—C3	1.3767 (18)	C8—O1	1.4381 (14)
C2—H2	0.9300	C8—H8A	0.9700
C3—C4	1.3795 (16)	C8—H8B	0.9700
C3—H3	0.9300	C9—C10	1.3912 (13)
C4—C5	1.3915 (15)	C9—C11	1.3938 (13)
C4—H4	0.9300	C9—N1	1.4183 (12)
C5—O1	1.3713 (11)	C10—C11^i^	1.3841 (14)
C5—C6	1.3912 (13)	C10—H10	0.9300
C6—C7	1.5113 (14)	C11—C10^i^	1.3841 (14)
C7—N1	1.4709 (12)	C11—H11	0.9300
			
C2—C1—C6	121.12 (10)	C6—C7—H7B	109.5
C2—C1—H1	119.4	H7A—C7—H7B	108.1
C6—C1—H1	119.4	N1—C8—O1	113.95 (9)
C3—C2—C1	119.55 (10)	N1—C8—H8A	108.8
C3—C2—H2	120.2	O1—C8—H8A	108.8
C1—C2—H2	120.2	N1—C8—H8B	108.8
C2—C3—C4	120.67 (10)	O1—C8—H8B	108.8
C2—C3—H3	119.7	H8A—C8—H8B	107.7
C4—C3—H3	119.7	C10—C9—C11	117.32 (9)
C3—C4—C5	119.50 (10)	C10—C9—N1	119.36 (8)
C3—C4—H4	120.3	C11—C9—N1	123.22 (8)
C5—C4—H4	120.3	C11^i^—C10—C9	121.98 (8)
O1—C5—C4	116.89 (9)	C11^i^—C10—H10	119.0
O1—C5—C6	122.47 (9)	C9—C10—H10	119.0
C4—C5—C6	120.63 (9)	C10^i^—C11—C9	120.70 (8)
C1—C6—C5	118.50 (9)	C10^i^—C11—H11	119.7
C1—C6—C7	121.66 (9)	C9—C11—H11	119.7
C5—C6—C7	119.83 (8)	C9—N1—C8	117.79 (8)
N1—C7—C6	110.80 (8)	C9—N1—C7	117.51 (8)
N1—C7—H7A	109.5	C8—N1—C7	108.90 (8)
C6—C7—H7A	109.5	C5—O1—C8	113.39 (8)
N1—C7—H7B	109.5		
			
C6—C1—C2—C3	−0.69 (17)	N1—C9—C10—C11^i^	−176.14 (8)
C1—C2—C3—C4	−0.61 (18)	C10—C9—C11—C10^i^	−0.28 (14)
C2—C3—C4—C5	0.91 (17)	N1—C9—C11—C10^i^	175.99 (8)
C3—C4—C5—O1	179.20 (10)	C10—C9—N1—C8	173.54 (9)
C3—C4—C5—C6	0.07 (16)	C11—C9—N1—C8	−2.67 (13)
C2—C1—C6—C5	1.63 (16)	C10—C9—N1—C7	−53.04 (11)
C2—C1—C6—C7	−179.07 (10)	C11—C9—N1—C7	130.75 (10)
O1—C5—C6—C1	179.60 (9)	O1—C8—N1—C9	71.79 (11)
C4—C5—C6—C1	−1.32 (15)	O1—C8—N1—C7	−65.29 (11)
O1—C5—C6—C7	0.29 (15)	C6—C7—N1—C9	−89.66 (10)
C4—C5—C6—C7	179.37 (9)	C6—C7—N1—C8	47.56 (11)
C1—C6—C7—N1	163.06 (9)	C4—C5—O1—C8	167.11 (9)
C5—C6—C7—N1	−17.65 (13)	C6—C5—O1—C8	−13.78 (14)
C11—C9—C10—C11^i^	0.29 (15)	N1—C8—O1—C5	47.26 (12)

Symmetry codes: (i) −*x*+2, −*y*+1, −*z*.

### Hydrogen-bond geometry (Å, °)

**Table:**

*D*—H···*A*	*D*—H	H···*A*	*D*···*A*	*D*—H···*A*
C8—H8A···O1^ii^	0.97	2.49	3.3469 (15)	147
C3—H3···Cg1^iii^	0.93	2.72	3.582 (13)	155
C3—H3···Cg1^iv^	0.93	2.72	3.582 (13)	155

Symmetry codes: (ii) −*x*+2, −*y*+2, −*z*; (iii) −*x*+1, *y*+1/2, −*z*−1/2; (iv) *x*+1, −*y*−1/2, *z*−1/2.
